# µSlicer for Programmable Multimaterial 3D Printing

**DOI:** 10.1002/advs.78364

**Published:** 2026-10-25

**Authors:** Daniel H. Meile, Berin Šeta, Md. Tusher Mollah, Yohan Jacquet, Rob Wolfs, Jon Spangenberg

**Affiliations:** ^1^ Department of Civil and Mechanical Engineering Technical University of Denmark Lyngby Denmark; ^2^ Department of the Built Environment Eindhoven University of Technology Eindhoven The Netherlands

**Keywords:** 3D printing, computational fluid dynamics, microstructure programming, raytracing

## Abstract

Nature rarely builds with uniform materials; instead, it achieves exceptional performance by arranging composition and structure across multiple length scales. Mimicking such architectures has become increasingly feasible with additive manufacturing, and especially with multimaterial 3D printing, where complex structures with spatially varying material composition can be fabricated. However, although 3D printing is widely recognized as a digital manufacturing method, multimaterial 3D printing remains only partially digitalized and still relies heavily on trial‐and‐error experimentation to identify printing strategies that produce the desired microstructures. To address this, we present the µSlicer that enables digital programming of internal multimaterial microstructures within individual printed strands. The µSlicer combines a numerical physics framework with an automatic signal generation module, allowing target microstructures to be translated into deposition signals for multimaterial extrusion. This approach fundamentally transforms slicing from purely geometric toolpath generation to microstructure programming, providing a route toward bioinspired, multifunctional, and digitally designed material architectures in extrusion‐based additive manufacturing.

## Graphical abstract

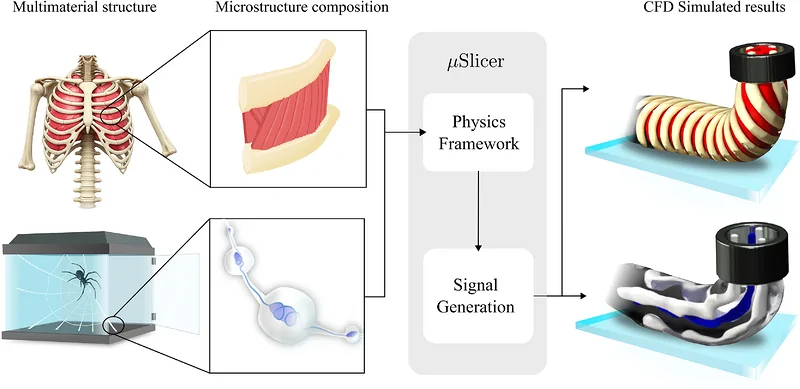

## 1 Introduction

Natural materials achieve exceptional combinations of mechanical performance and multifunctionality through finely tuned material architectures embedded within their fundamental building blocks. In many biological systems, individual structural elements exhibit internal heterogeneity, anisotropy, and functional separation, such that spatially varying composition within a single element directly governs macroscopic behavior. Notable examples include spider silk with distinct core–shell protein architectures [1], plant vascular elements combining hydrological transport and mechanical support [2] within concentric structures, and mineralized biological tissues such as bone osteons or squid beaks that exhibit pronounced gradients in composition and stiffness [3]. These element‐level architectures enable efficient load transfer, damage tolerance, and functional integration that are difficult to reproduce using homogeneous constituents, and span a wide range of length scales, from microscopic to macroscopic, depending on the function they serve [4].

Replicating these architectures in engineered systems would require material control at scales comparable to the relevant natural building blocks. In voxel‐based multimaterial approaches, this implies discretising the entire part volume at a very fine spatial resolution, using voxels with characteristic dimensions comparable to the nozzle diameter, which becomes physically impractical when fabricating large structures. Consequently, even though additive manufacturing enables complex geometries and spatial material placement, material heterogeneity is typically introduced at the layer [5, 6] or component scale [7], while individual deposition paths are treated as materially uniform. As a result, geometric complexity and material complexity remain largely decoupled at the strand level, limiting the translation of bioinspired material concepts. Recent advances in extrusion‐based additive manufacturing demonstrate that material heterogeneity within a single printed strand is technically feasible. Multimaterial extrusion strategies [8], including core–shell [9, 10], coaxial [11], and sequential [12] enable spatially resolved material distributions along and across individual filaments.

These capabilities have primarily been demonstrated using highly tailored, application‐specific material systems that rely on complex rheology, chemistry, or processing conditions to achieve stable multimaterial extrusion [13, 14, 15]. As a consequence, multimaterial strand architectures are often realized as isolated demonstrations rather than as digitally programmable design entities. This limits their systematic use for hierarchical and hybrid material architectures found in nature and targeted in advanced engineering and biomedical applications.

Here, we move beyond material‐specific, trial‐and‐error experimental approaches by introducing a physics‐informed µSlicer (see Figure 1) for programming internal strand‐level microstructures across multiple material families and microstructure designs. In a broad sense, the µSlicer can be understood as the microstructural analogue of conventional slicing: whereas traditional slicing algorithms convert geometric models into toolpaths that define where material is deposited, the µSlicer ensures that internal material architecture matches predefined target within each strand. Rather than treating printed strands as simple lines that fill a geometry, we treat them as designed building blocks containing programmed multimaterial microstructures. This shifts the focus from designing shapes to designing strands, in which the internal composition of each deposited strand becomes a programmable design variable. The µSlicer therefore slices strands to obtain microstructure within them rather than slicing 3D objects into layers. Nevertheless, 1D strands are fundamental building blocks of 3D structures, and enabling control in 1D strands inherently unlocks this capability in 3D structures. Material complexity can thus be embedded at the same level at which geometry is traditionally controlled, enabling bioinspired materials and structures whose internal architecture is digitally specified. While the approach is general across multiple material families and extrusion strategies, the current work fully demonstrates its application through rotational multimaterial extrusion.

**FIGURE 1:**
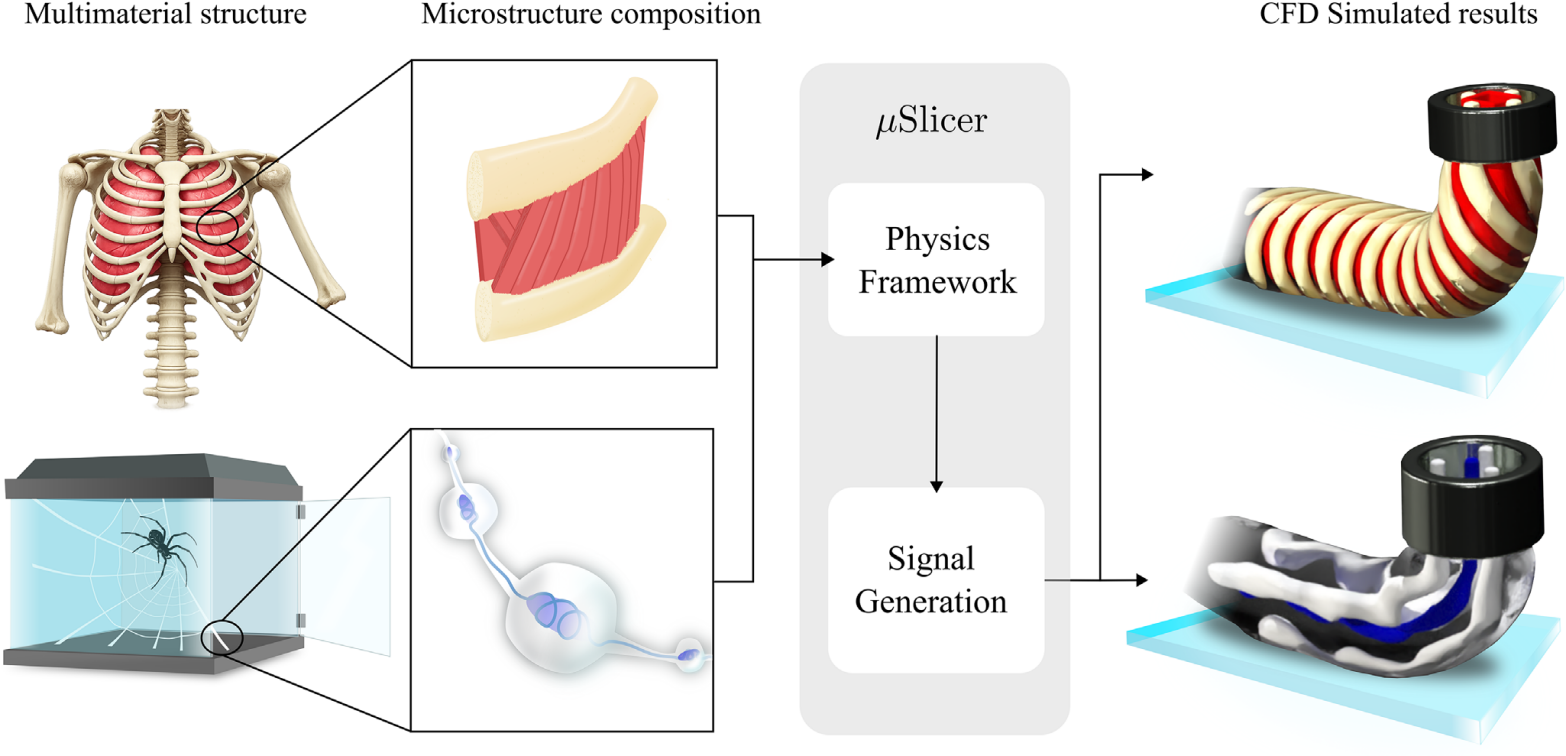
Digital‐driven design of natural‐inspired systems, such as rib cage and spider silk. The details on the obtained multimaterial structures on the right are available in Methods, *Bioinspired multimaterial structures*.

The µSlicer consists of two coupled components: a physics framework and a signal generation module. The physics framework predicts how materials flow and form microstructures during extrusion and deposition, establishing a link between the flow profile and the resulting microstructure. Based on material flow behavior and a target microstructure, the signal generation module produces the deposition signals, such as nozzle rotation and volumetric flow rate, required for physical realization. Below, we describe these two components, followed by demonstration cases.

## 2 Physics Framework ‐ Numerical Validation

The integral part of the µSlicer is the ability to accurately predict microstructure morphology within 3D‐printed strands. In order to do so, we developed a physics framework that is represented by a computational fluid dynamics (CFD) model and validated it against the benchmark experiments on rotational multimaterial 3D printing (RM‐3DP) [16]. This study presents a particularly complex validation case: while multimaterial deposition is often achieved by modifying nozzle geometry or by adjusting outlet flow rates, Larson et al. additionally introduced complex, time‐dependent rotational motion of both inclined and non‐inclined nozzles [16]. Consequently, our validation required reproducing not only the nozzle geometry but also the full kinematics of the rotational printing process.

As a first step, the experimentally reported rheological data [16] were fitted to a power‐law constitutive model to capture the shear‐thinning behavior of the fluid viscosity (more details in Methods, *Rheology*). The viscoelastic response was modeled using the Giesekus constitutive equations, with the storage and loss moduli taken from the same experimental dataset to ensure consistent and comparable material properties across all simulations in the numerical model validation step.

Moreover, to enable direct comparison with the experiments, we adopted the same non‐dimensional printing parameters: the non‐dimensional rotational rate $\omega^{*}=\left(\omega R\right)/V$, the non‐dimensional gap height $h^{*}=h/\left(2R\right)$, and the non‐dimensional flow rate $Q^{*}=Q/\left(V\pi R^{2}\right)$, where Q denotes the total outlet flow rate (see Figure 2a). In all simulations, the translational printing direction $\theta_{D}$ was fixed at zero, while various extrusion angles $\theta_{P}$ were explored. The nozzle geometry was constructed to replicate the idealized configuration used by Larson et al. [16] to compute flow rates (Figure 2a,b), consisting of three outlets: one for the bulk (transparent) material and two internal outlets (blue and red). For computational efficiency, minor geometric features, such as the curvature of the outlet sidewalls that were used in the experiments [16], were omitted.

**FIGURE 2:**
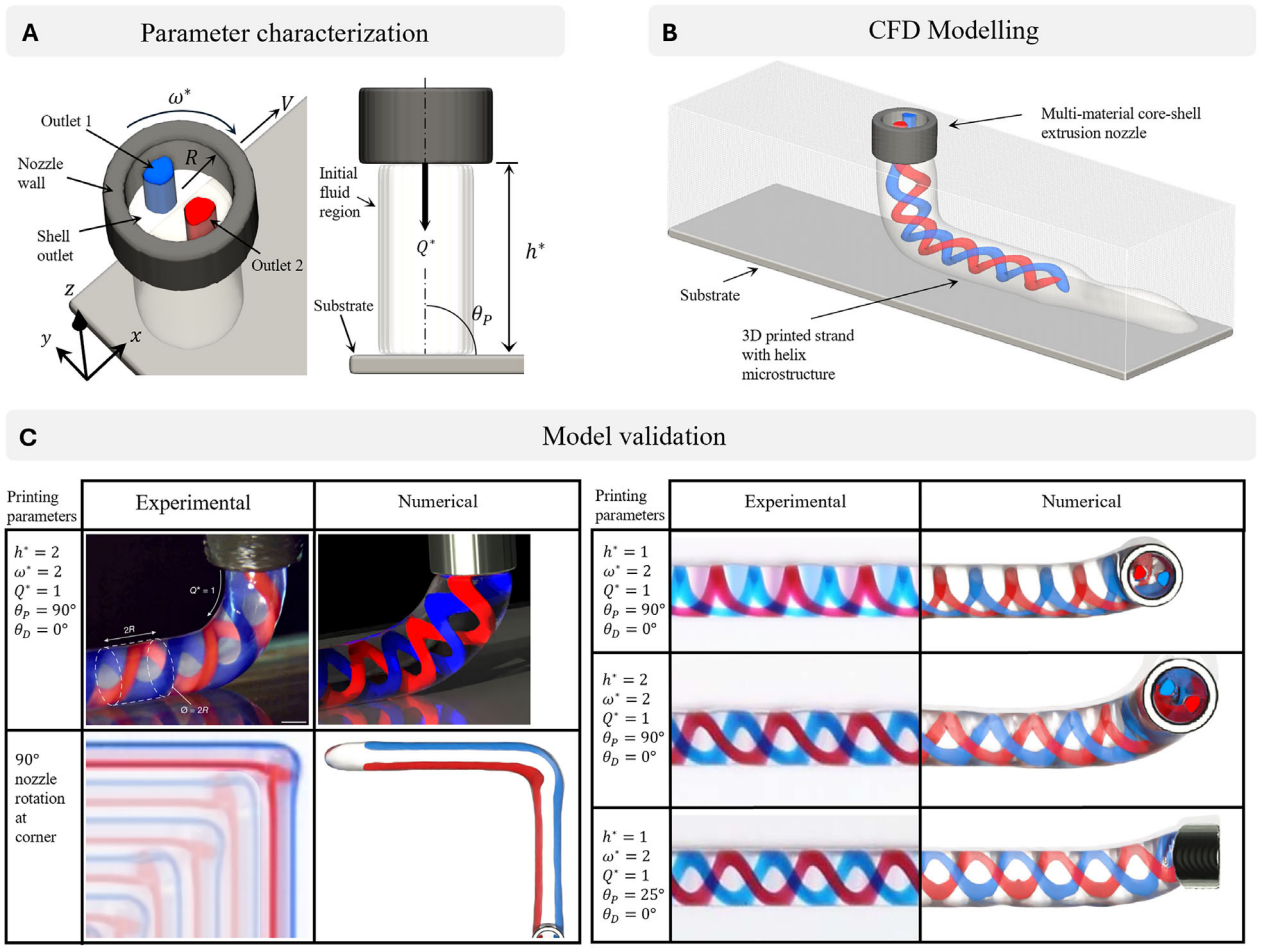
Validation of numerical model. (a) Nozzle design, non‐dimensional parameters and boundary conditions. (b) Numerical model in the process of depositing dual‐core helical filaments with induced rotation rate and parameters ($h^{*}=2,w^{*}=2,Q^{*}=1,\theta_{P}=90,\theta_{D}=0$) (c) Model validation for different cases from experimental study on multimaterial internal structure [16].

As shown in Figure 2c, the model reproduces the experimentally observed microstructural patterns across a broad range of printing parameters with high fidelity (for quantitative measurements refer to Methods, Figure 9). The agreement holds for cases involving pure rotation, combined rotation and inclination, and even during a discrete 90‐degree rotation while printing a corner, ensuring continuous deposition of the red and blue materials on their designated sides of the strand. As part of our validation, it was necessary to account for optical refraction, as visual inspection of microstructures in transparent materials can significantly distort their apparent morphology, particularly at the subvoxel scale (i.e., sub‐diameter scale in extrusion‐based 3D printing). To enable meaningful comparison between simulations and experiments, CFD‐predicted microstructures from the µSlicer were post‐processed using physics‐based ray tracing, revealing systematic apparent shifts and magnification effects with increasing refractive index. Details on the refraction effects and the applied methodology are provided in Methods, *Raytracing of microstructure: what you see is not what you get*.

**FIGURE 3:**
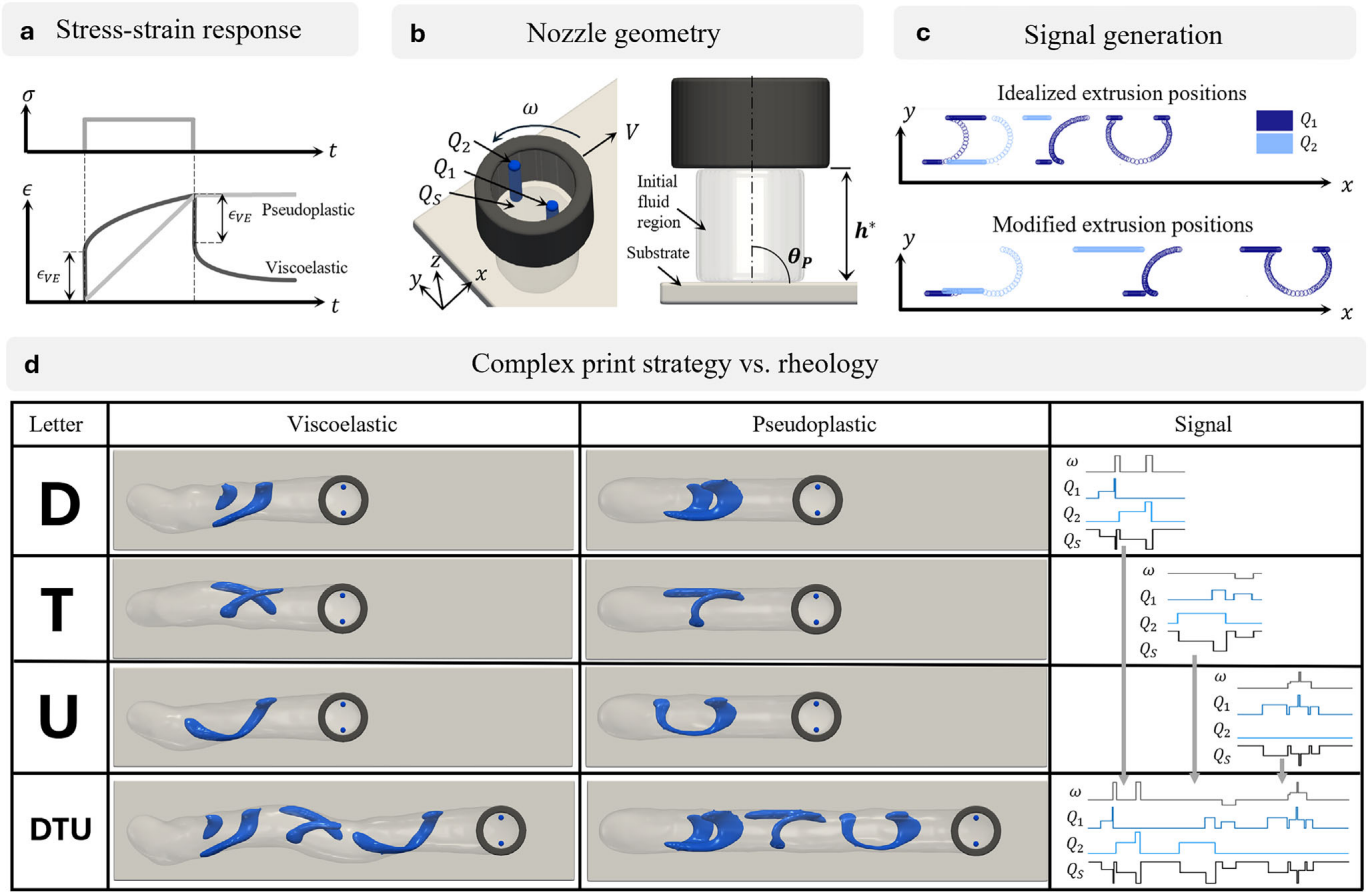
Rheological effects on microstructure. (a) Strain–stress response of the viscoelastic and pseudoplastic model. (b) Nozzle geometry, with the initial and boundary conditions, as well as position of the outlets (in blue). (c) Manual signal generation in Matlab. The top row shows the idealized trajectory that would produce the “DTU” microstructure when viewed from above, while the bottom row shows a manually modified signal that more closely reproduces the “DTU” microstructure in the numerical simulation. d) “DTU” microstructure embedded in transparent fluid for both viscoelastic and pseudoplastic material, along with the detailed signal of flow rate of each outlet and the bulk material, as well as rotation.

**FIGURE 4:**
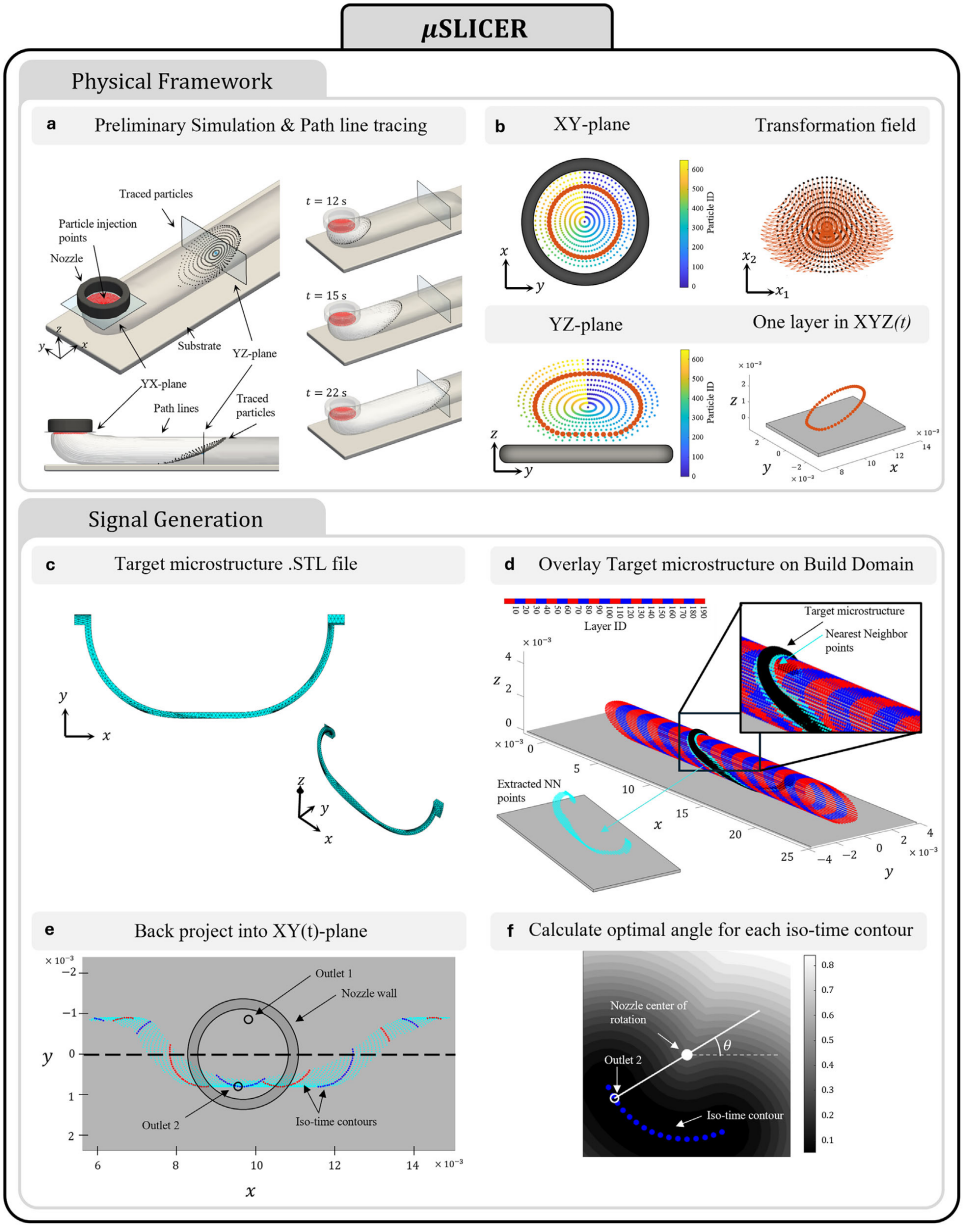
Different components and prerequisites for µSlicer. (a) Simulation of strand deposition and path line tracking for passive injected particles at the outlet. (b) Mapping of points between the cross‐section of the outlet and the cross‐section of the strand. (c) Targeted microstructure (d) Projection of targeted microstructure onto time‐resolved flow field. (e) Back projection into nozzle plane (f) Optimal angle calculation.

**FIGURE 5:**
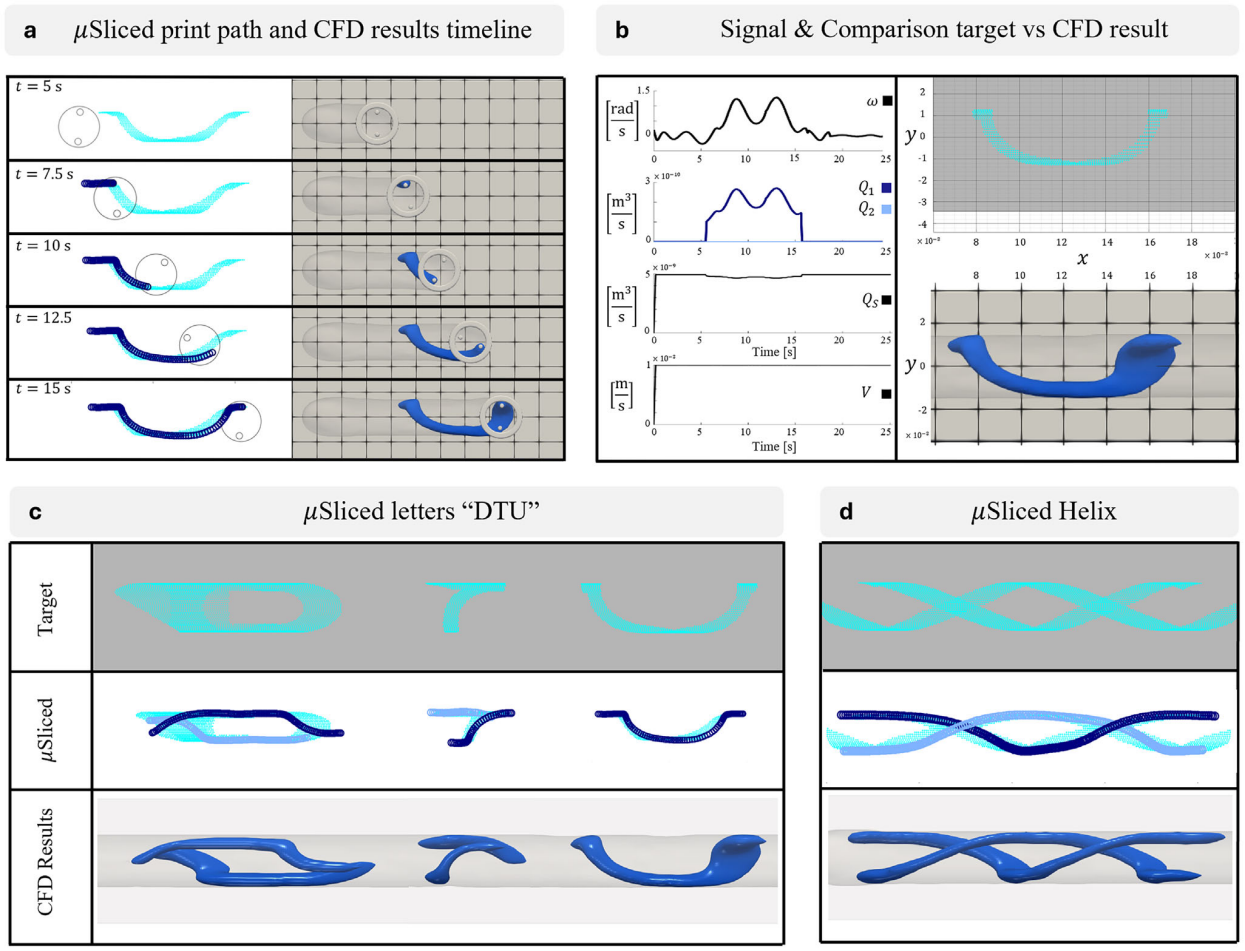
Demonstration of µSlicer. (a) µSlicer input on how to write letter U. (b) All the relevant parameters subjected to optimization (rotation speed, extrusion rates of the bulk material, and two outlets). (c) Results from µSlicer on “DTU” pattern (d) Results from µSlicer on helical patterns similar to Larson et al. [16].

**FIGURE 6:**
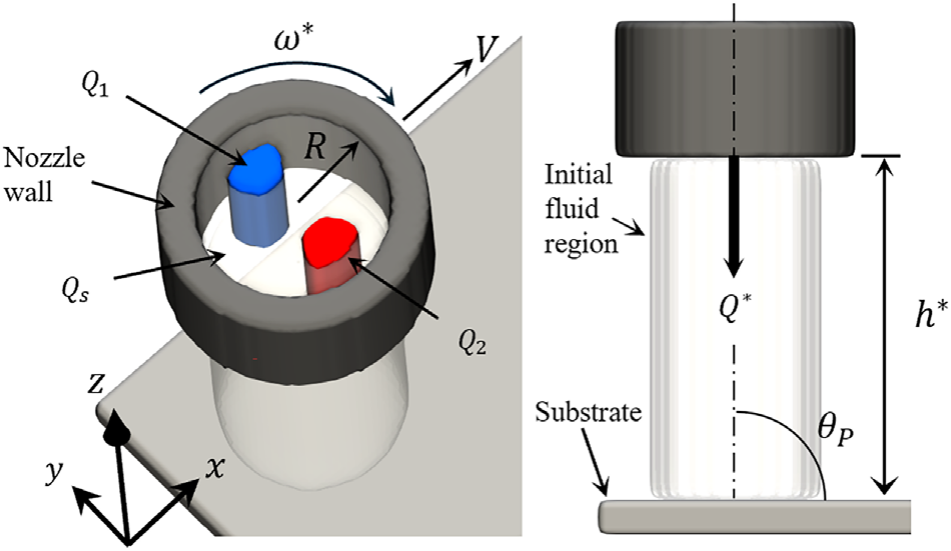
Idealized nozzle geometry based on Larson et al. [16].

**FIGURE 7:**
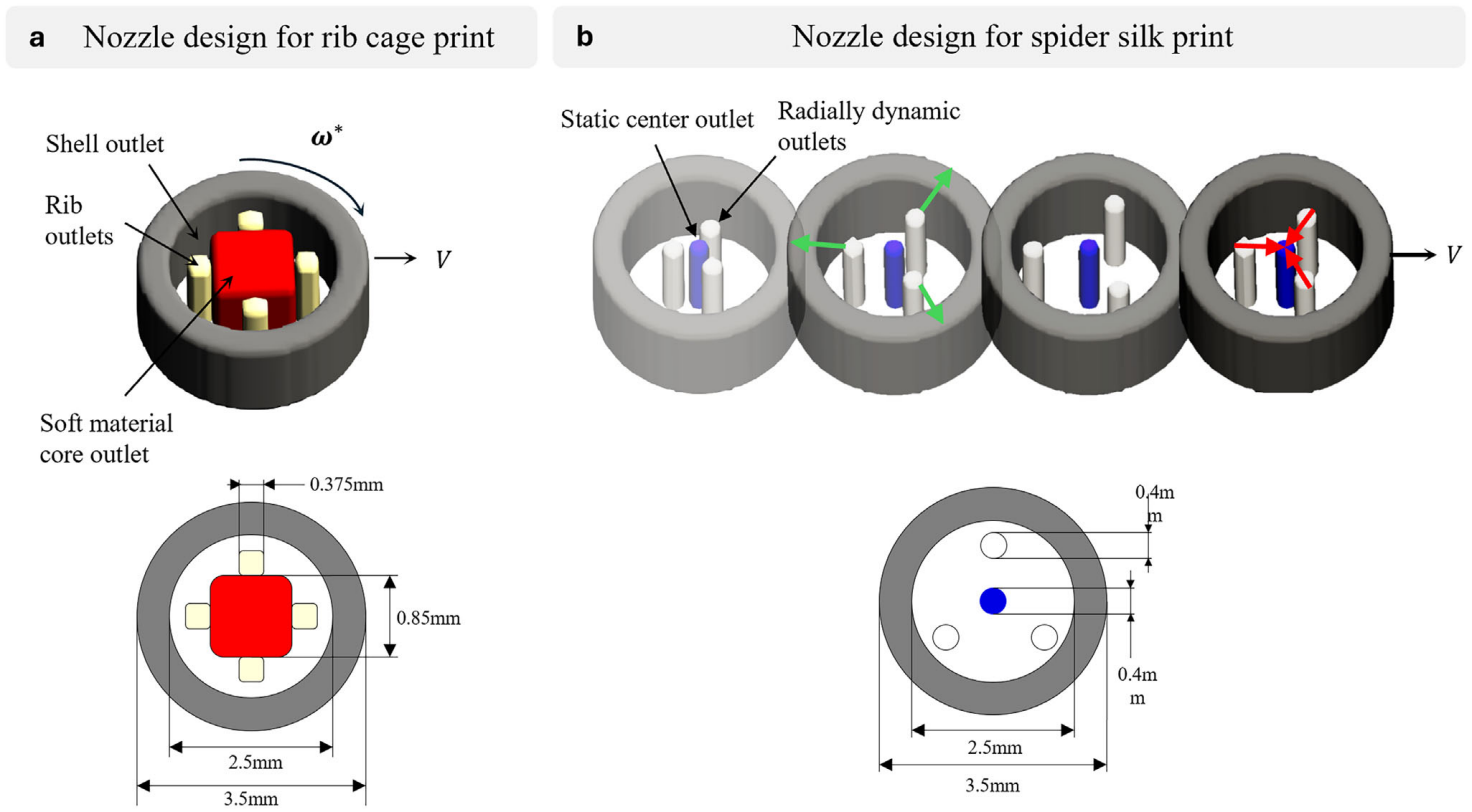
Design of complex nozzles used in (a) rib cage, and (b) spider silk multimaterial structure prints.

**FIGURE 8:**
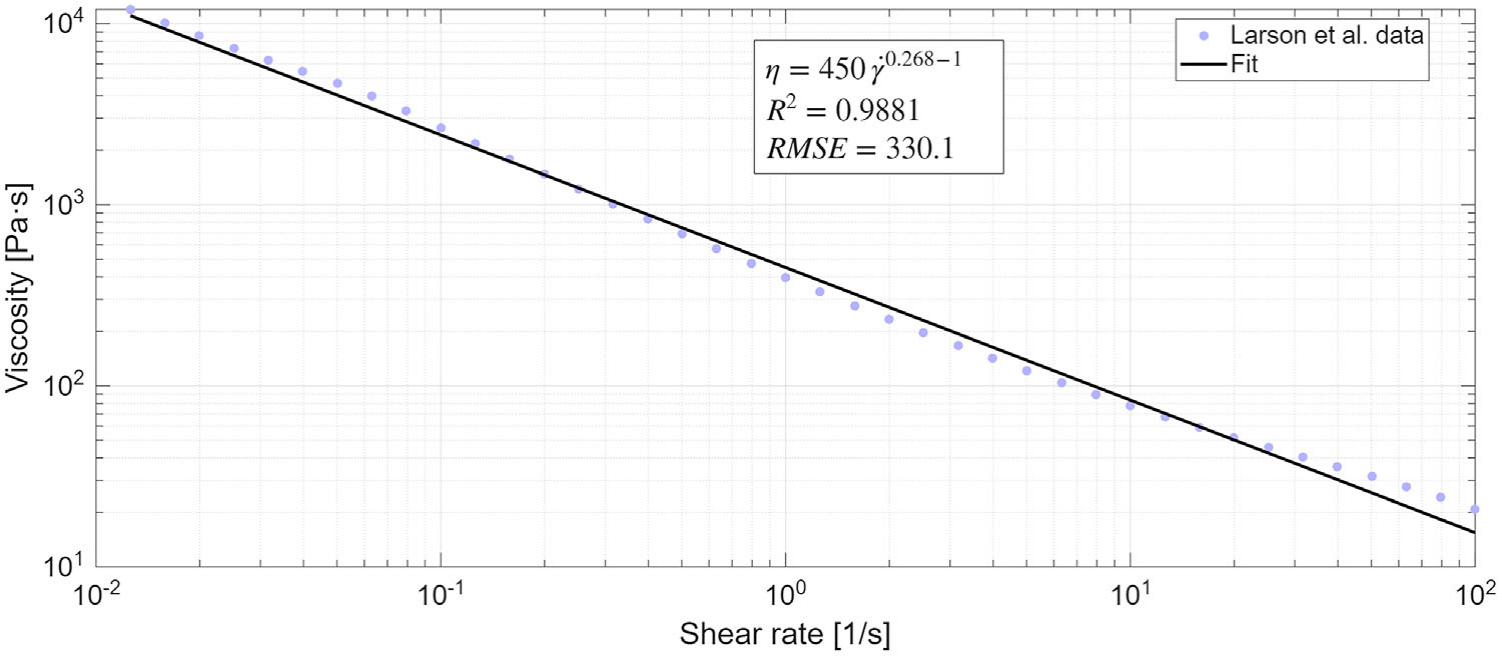
Fitted data from Larson et al. [16] to Power‐law model.

**FIGURE 9:**
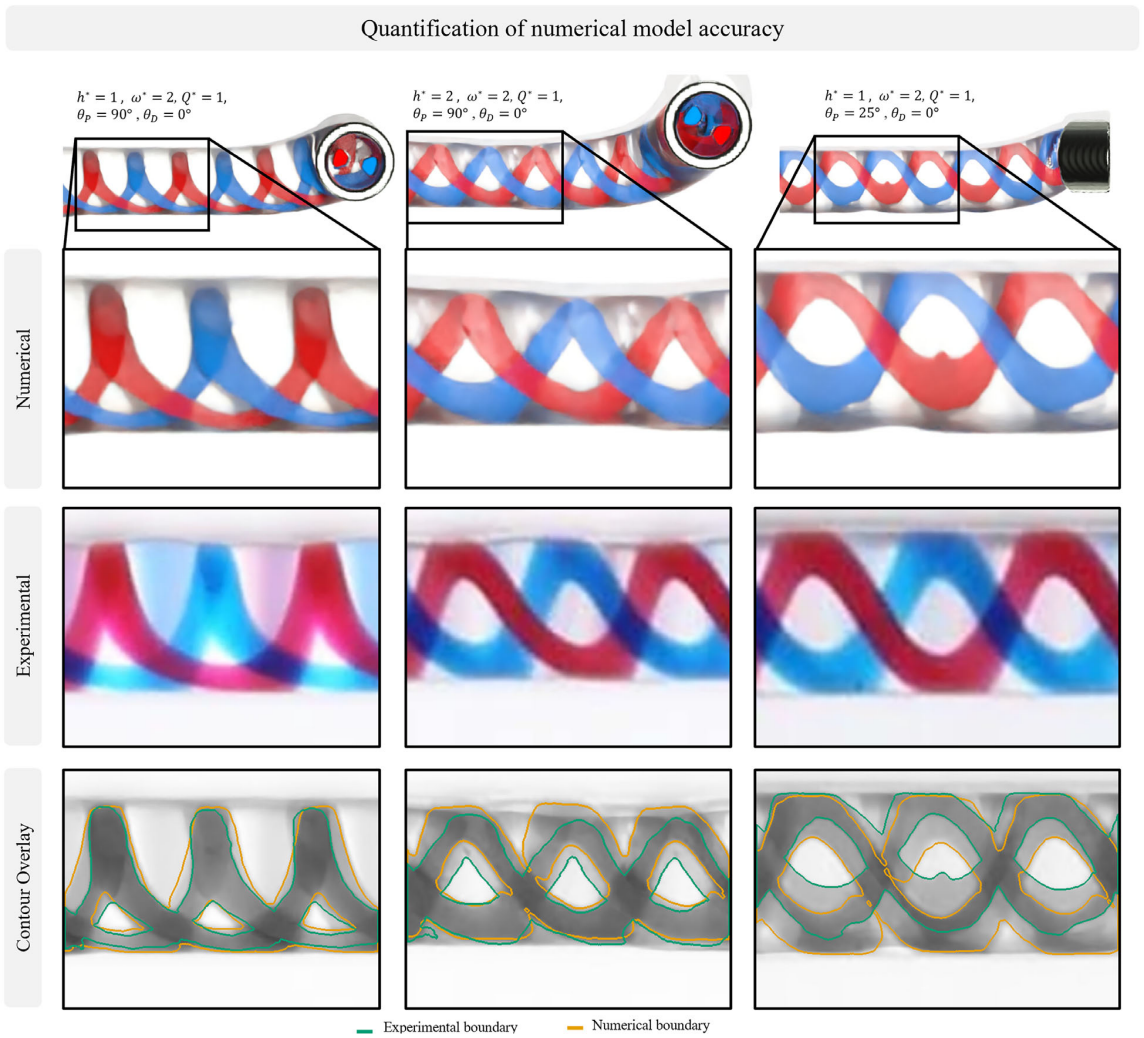
Computed Dice and IoU metrics for the helical structures by comparing the numerical and experimental results [16], indicating a high degree of overlap between the two. It should be noted that these results may be affected by differences in lighting conditions, the refractive index of the transparent bulk material, and the observation angle.

With this validated model in place, optimization of nozzle design, printing parameters, and fully digital programming of microstructure becomes possible, moving beyond empirical trial‐and‐error approaches toward predictive, design‐driven control of microstructural architecture.

### 2.1 Physics Framework ‐ Rheological Considerations

The ability to program internal microstructures through controlled multimaterial extrusion depends not only on the prescribed nozzle motion and extrusion sequence, but also on how the material responds to deformation during deposition. In particular, the mapping between an input control signal ($Q_{1},Q_{2},Q_{S},\omega$, see Figure 3) and the resulting microstructure is strongly influenced by the rheological behavior of the extruded material. To examine this effect, we compare viscoelastic and pseudoplastic (shear‐thinning) fluid models, representing two common rheological classes in extrusion‐based 3D printing and spanning material behaviors from elastomeric silicone‐based inks such as PDMS formulations to shear‐thinning materials such as pastes, gels, and bioinks. These rheological classes govern how programmed material trajectories are translated into deposited microstructures, and thus directly impact the ability to predict and control microstructure formation within the µSlicer framework. Viscoelastic fluids exhibit an instantaneous elastic strain response followed by a time‐dependent flow and stress relaxation (Figure 3a). When subjected to a constant shear stress σ, the fluid initially stores elastic strain $\varepsilon_{\mathrm{VE}}$, determined by the storage modulus 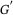, after which it begins to flow according to a nonlinear creep while gradually relaxing its internal stresses. Upon removal of the applied stress, the stored elastic strain is released instantaneously, and the fluid proceeds to relax toward its equilibrium state. In contrast, pseudoplastic fluids exhibit a viscosity that decreases with increasing shear rate and do not store elastic energy. As a consequence, pseudoplastic flow exhibits no shear‐history dependence, and the internal material position responds directly to the instantaneous applied shear rate, making microstructure placement more predictable.

With the validated microstructure prediction model, we examined whether controlled multimaterial extrusion and nozzle rotation could generate complex, user‐defined microstructures such as readable letters embedded within a printed strand using the viscoelastic and pseudoplastic rheological models. The materials properties for viscoelastic materials refer to experimental values from Larson et al., while pseudoplastic materials use the power‐law without an elastic component. To enable such programmability, the nozzle configuration was slightly modified from the fan‐shaped geometry used by Larson et al. and replaced with circular outlets: two inner outlets ($Q_{1}$ and $Q_{2}$) extruding blue‐dyed, and an outer annular outlet ($Q_{s}$) extruding transparent material (Figure 3b). In all simulations, the non‐dimensional gap height and flow rate were fixed at $h^{*}=1$ and $Q^{*}=1$, respectively, with a constant translation speed of $V=0.001\;m\;s^{-1}$. During rotation, the outlet flow rates were coupled proportionally to the rotational speed.

The programmed microstructure was achieved through a manually repeated simulation‐correction cycle. As an initial step, an idealized material placement trajectory was prescribed, as shown in Figure 3c, by defining a sequence of alternating nozzle rotations combined with timed extrusion from $Q_{1}$ and $Q_{2}$. The initial trajectory was generated in MATLAB such that, under a purely kinematic mapping between the prescribed input signal and the deposited material, the intended microstructure would reproduce the letters “DTU”, when viewed from above. In practice, however, the idealized trajectory did not directly produce the expected deposited pattern. The final material placement was affected by material deformation during deposition and by inertial effects arising from the relatively high nozzle rotational velocities. To account for these effects, the input signals for nozzle rotation and material extrusion were manually modified, and the simulations were repeated until the resulting microstructure more closely matched the targeted one. This is illustrated by the formation of letter “D”. In the input signal provided to the CFD solver, the modified extrusion appears as a horizontal segment at the beginning of the deposition process (signals in Figure 3c). During deposition, however, the material is subjected to strong counter‐clockwise shear from the rapidly rotating nozzle, which displaces it into the desired curved shape, at least for the pseudoplastic model. This example highlights that the final deposited geometry did not follow directly from the idealized trajectory, but from the interaction between the prescribed signal and the material response. In the following section, this manual repeated simulation‐correction cycle is replaced by automatic signal generation within the µSlicer framework.

Since pseudoplastic fluids respond directly to the applied shear, their behavior is more predictable and less affected by inertial forces caused by nozzle rotation. Consequently, the simulation results show that with the modified signal readable letter‐shaped microstructures are readily achievable for the pseudoplastic model (see Figure 3d). In contrast, significant deviations arise in the viscoelastic model, including transverse drift of the entire strand and loss of geometric fidelity. These deviations originate from the creeping‐flow behavior and the accumulation of elastic strain, both of which cause the material interface to lag behind the programmed trajectory. This not only affects the overall strand outline but also the internal microstructure. Elastic strains are stored differently across the strand, leading to spatially varying creeping behavior. As a result, the viscoelastic effects act unevenly on different regions of the microstructure, manifesting in our case as distortions in the letter formations. Nevertheless, as the relaxation time in the viscoelastic model was reduced, the distortions became progressively less pronounced. This effect was systematically investigated in Methods, *Effect of relaxation time on patterning*, illustrating that some viscoelastic fluids may be easier to program than others depending on their rheological properties.

Ultimately, the reliance on manually repeated simulation‐correction cycles to obtain a signal highlighted the need for a more systematic approach. We examined whether the extrusion positions could be determined a priori by incorporating the flow response into the signal generation itself. Such an approach would enable a physics‐informed µSlicer to generate a signal capable of producing microstructure deposition paths based solely on the anticipated flow field, eliminating the iterative tuning that currently limits programmability.

## 3 Signal Generation

The previous sections established a numerical framework for predicting microstructure formations from nozzle design, printing parameters, rheological properties, and kinematics such as rotation and inclination. Using this framework, we demonstrated the reproduction of letter‐shaped microstructures with a rotating nozzle and independently activated outlets. We now introduce the second component of the µSlicer: the automatic signal generation module that, based on information from the physical framework, determines the printing strategy required to realize a prescribed microstructure. The procedure begins with a preliminary single‐fluid CFD simulation to characterize the flow field and determine how material originating from the nozzle is transformed within the deposited strand. Using this flow information together with the target microstructure provided as a 3D model in STL format, the µSlicer identifies the required target regions and generates the corresponding printing signal. The complete workflow is illustrated in Figure 4 and described in detail below.

The flow‐profile calculation, shown in Figure 4a,b, involves simulating the strand deposition using pseudoplastic material properties, since these facilitate easier programmability, as demonstrated in the previous section. After the flow field is obtained, 650 passive particles are introduced into the fluid in a single cross‐section in the nozzle, and their pathlines are analyzed by relating their initial positions inside the nozzle to their final positions in the deposited strand (see the transformation field in Figure 4b). Each particle injection point is assigned a unique ID, allowing us to track its mapping through the flow. This approach provides a way to associate regions of the nozzle with corresponding locations in the final microstructure. For example, a set of particle points with equal radial position (the red circle in Figure 4b) deforms into an oval shape in the strand, illustrating how the nozzle geometry and flow affect material placement. By analogy, outlets such as those used in previous sections could be treated as groups of particles whose trajectories can be mapped into the strand.

After establishing the mapping at a single time step, we extended the analysis to the full time‐resolved flow field onto which the target microstructure is projected (see Figure 4c,d). This allowed us to determine how a time‐dependent extrusion signal is spatially transformed within the strand. As illustrated in Figure 4d, the alternating blue and red markers show the positions of the same set of particles at different times during deposition. This mapping is crucial because not all regions of the nozzle deposit material along the same vertical line. As particles are carried by the flow along paths of different lengths and velocities, they reach the same cross‐section of the strand at different times, or equivalently, different cross‐sections at the same time. Capturing the temporal offset is essential for constructing a slicer that generates time‐dependent signals capable of targeting specific regions of the deposited microstructure [17].

Once the target microstructure is imported as an STL file (here the letter “U” in Figure 4c), its points are projected onto the time‐resolved particle positions from Figure 4d. Intersections between the STL geometry and the mapped particle trajectories are then identified, and the corresponding particles are designated as the targeted points in the generated signal (see Figure 4b). The targeted points were generated using a Nearest Neighbor (NN) algorithm (cf. Methods, *µ Slicer Optimization Loop*). After ensuring that the extracted NN points (Figure 4d) adequately represent the intended STL geometry (Figure 4c), these points are projected back onto the nozzle x−y plane. As shown in Figure 4e, these back‐projected points are formed from a set of iso‐time contours (lines representing particles extruded simultaneously), indicated by red lines, from which a single representative point per contour is matched by one of the material outlets and selected to generate the extrusion signal. Starting from the first selected point, the optimal nozzle rotation angle is then computed to follow the local geometry of the target microstructure (Figure 4f, see Methods, *Selection of the initial target point*). Repeating this procedure across successive iso‐time contours yields a sequence of optimal nozzle rotation angles and which extrusion outlet is active at each timestep, enabling full reconstruction of the signal required to 3D print the target microstructure using rotational multimaterial 3D printing.

## 4 µSlicer Demonstration

The capabilities and performance of the µSlicer are demonstrated in Figure 5a–c, where complex microstructures representing the letters “D”, “T”, and “U”, as well as a helical structure (cf. Figure 5d) previously studied by Larson et al., are successfully reconstructed. These are independent, automated demonstrators, in contrast to those presented in Section 2.1, where the signal was manually generated. This marks an important step toward fully automated microstructure programming, showing that complex three‐dimensional material distributions can be generated directly from a target geometry rather than through manual tuning of the printing parameters. The functionality and workflow of the µSlicer is exemplified through the formation of the letter “U”. In Figure 5a, the automatically generated µSlicer signal is shown side by side with the corresponding CFD simulation result, demonstrating how the slicer output translates into the reconstructed material distribution. In this case, only a single outlet (dark blue) was required to reconstruct the target geometry, as it was sufficient for capturing the letter shape. The selection between using one or multiple outlets is controlled through a weighting factor within the µSlicer, and for the letter “U”, a single outlet was preferred due to the simplicity of the geometry.

For each target geometry, the µSlicer provides the necessary inputs to the CFD solver, including the rotational speed ω, activation state, and the flow rates of the individual outlets $Q_{1}$ and $Q_{2}$, and the bulk material flow rate $Q_{s}$. The translational velocity of the nozzle is also prescribed by the slicer and is kept constant in all cases, as shown in Figure 5b. Since each outlet can target only one point at a given printing time, the µSlicer must assign a single target point from each iso‐time contour to each outlet. This makes the initialization particularly challenging because multiple target points may be reachable at the same printing time, while only one can be selected by each outlet. To reduce this ambiguity, the input geometries were designed to impose favorable initial conditions and to guide the algorithm toward a well‐defined starting point of the microstructure. For instance, the µSliced “U” signal shown in Figure 5b was generated from a twisted tubular geometry wrapping around the strand wall. This geometry promotes a specific outlet position (at a specific time) as an initial condition, which subsequently enables the same outlet to be used throughout the print (with adjusted weighting factors). While the letter “U” represents the simplest case, requiring only one outlet, more complex geometries require coordinated use of both outlets, as demonstrated by the reconstruction of the letters “D” and “T” in Figure 5c and the helical structure in Figure 5d.

## 5 Discussion

The µSlicer combines a physics framework, validated against benchmark experiments on rotational multimaterial 3D printing [16], with an automatic signal generation module. The physics framework predicts how nozzle configuration, printing parameters, material rheology, and kinematics influence the resulting strand microstructure, while the signal generation module maps target geometries onto time‐resolved particle trajectories and determines the required nozzle rotation, outlet activation, and material flow signals. This workflow was demonstrated through the generation of internal architectures including the letters “D”, “T”, and “U”, as well as a helical microstructure, showing that deposition signals can be digitally programmed to obtain desired strand‐level microstructures rather than being identified through manually repeated trial‐and‐error experimental procedures. The results further show that rheology plays a decisive role in microstructure programmability: pseudoplastic materials are more directly programmable, whereas viscoelastic materials can exhibit reduced geometric fidelity due to elastic strain accumulation and relaxation.

At the same time, the current implementation has a few limitations. The automatic signal generation algorithm is based on a transformation field obtained from pure extrusion under a fixed set of printing parameters. As a result, inertial effects caused by complex nozzle motions are neglected in the present µSlicer formulation, even though they may influence material placement. However, this limitation reflects the current implementation rather than the underlying methodology. If the transformation field is generated using simulations that include these inertial effects, the same framework could incorporate their influence directly into the generated signals. Another limitation is that the present optimization strategy still benefits from human guidance in the design of suitable target geometries and in the selection of the initial target point. Future work should therefore focus on improving the optimization algorithm to enable more autonomous initialization while maintaining computational efficiency.

It is important to note that the µSlicer is fundamentally a material placement tool, not a mechanical property prediction tool. The framework enables users to digitally specify and realize target strand‐level microstructure geometries; however, the connection between these geometries and resulting mechanical or functional properties remains the responsibility of the user. Users must independently determine which microstructure designs achieve their desired performance targets through mechanical analysis or prior knowledge; the µSlicer then provides the signal generation capability to realize those microstructures.

In this way, the µSlicer expands the role of slicing from geometric toolpath generation to digital microstructure programming. Although the present work focuses on multimaterial strand architectures, the methodology is not limited to material switching and could be extended to other types of microstructure slicing, including the spatial control of filler particles, fibers, flakes, or anisotropic inclusions. By enabling internal strand architectures to be digitally designed and generated, the µSlicer establishes a new level of control over the fundamental building blocks of printed structures and reframes extrusion‐based additive manufacturing as a platform for programmable material architecture. This transition from trial‐and‐error printing to predictable and programmable functionality marks a critical step toward moving architected materials beyond the laboratory and into reliable applications in biomedicine, soft robotics, and responsive structural systems.

## 6 Methods

### 6.1 Nozzle Designs

The nozzle geometry used in the validation simulations was modeled to replicate the ideal configuration used by Larson et al. [16], shown schematically in Figure 6. This nozzle includes three outlets: one for the bulk transparent PDMS material ($Q_{s}$) and two for the inner core materials ($Q_{1}$ & $Q_{2}$). To reduce computational cost, the intricate internal sidewalls of the nozzle used in the experiments were simplified in the numerical model. The nozzle was placed above a flat substrate within a defined computational domain, allowing for the simulation of deposition under comparable conditions. For the µSliced simulations, the nozzle was slightly modified by replacing the $Q_{1}$ and $Q_{2}$ outlets with circular cross‐sections with a radius of r=0.2 mm instead of the original fan‐shaped geometry.

### 6.2 Bioinspired Multimaterial Structures

The rib‐cage‐ and spider‐silk‐inspired structures from Figure 1 were generated using the physical framework, in which both the nozzle design and the prescribed nozzle motion were modified.

For the rib‐cage‐inspired structure (see Figure 7a), the nozzle consisted of four small outlets depositing a white rigid material, mimicking bone‐like features, and one central outlet depositing a red soft material, mimicking soft tissue. These outlets were embedded within a transparent bulk material, which was deposited through a separate outlet. During printing, the entire nozzle was rotated, following the same principle used in the validation cases presented in the manuscript.

For the spider‐silk‐inspired structure, the nozzle contained four small outlets embedded within a transparent bulk medium deposited separately. Three of the outlets deposited white material, while the fourth, central outlet deposited blue material. Unlike the previous cases, no global nozzle rotation was applied. Instead, the three white‐material outlets moved dynamically within the larger nozzle during printing, resulting in time‐dependent outlet positions, as shown in Figure 7b. This demonstrates that the proposed physical framework is not limited to a specific nozzle design or motion strategy, but can be applied to different modes of control in multimaterial 3D printing.

### 6.3 Non‐Dimensional Printing Parameters

To ensure comparability between the CFD model and the experimental setup of Larson et al. [16], the same non‐dimensional printing parameters were employed. These are defined as:

(1) $$\omega^{*}=\frac{\omega R}{V},\;h^{*}=\frac{h}{2R},\;Q^{*}=\frac{Q}{V\pi R^{2}},$$

where ω is the rotational speed, R is the nozzle radius, V is the translational velocity, h is the gap height, and Q is the total volumetric flow rate through all outlets. The flow rate Q represents the sum of the outlet flow rates, $Q=Q_{s}+Q_{1}+Q_{2}$, corresponding to the bulk and core channels, respectively.

### 6.4 Rheology

The complex rheology of the polydimethylsiloxane (PDMS) material was based on the experimental measurements reported by Larson et al. [16]. In their work, detailed rheological data were published on polydimethylsiloxane (PDMS), which revealed viscoelastic characteristics. To account for the shear‐thinning behavior of the PDMS material, the shear‐rate dependency of the apparent viscosity, $\mu_{\text{app}}$, was characterized by fitting the viscosity shear rate data (Figure 8) to a Power‐law model of the form:

(2) $$\mu_{app}\left(\dot{\gamma }\right)=K\;{\dot{\gamma }}^{\;n-1}$$

By which, K is the consistency index, n the Power‐law index, and $\dot{\gamma }$ the shear rate. From the fitted data, the aforementioned Power‐law model parameters was found and can be seen listed in Table 1.

**TABLE 1:** Rheological parameters used in the numerical simulations.

Variable	Symbol	Unit	Value
Consistency index	K	$\mathrm{Pa}\cdot s^{n}$	450
Power‐law index	n	—	0.268
Critical shear rate	$\dot{\gamma }$	$s^{-1}$	0.01
Storage modulus	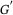	Pa	28000
Relaxation time	λ	s	0.3

### 6.5 CFD Model

The CFD model was developed in the commercial software flow‐3d, version V2022R1 [18], and assumed the flow of PDMS material to be isothermal, transient, incompressible, and laminar, by which the governing equations, that were discretized using the Finite Volume Method (FVM), were described by the continuity and momentum equations.

(3) ∇·u=0,

(4) $$\rho \left(\frac{\partial u}{\partial t}+u\cdot \nabla u\right)=\rho g-\nabla p+\nabla \cdot S_{T}$$

where u is the velocity field, $\rho =1120\mathrm{kg}/m^{3}$ is the constant density, p is the pressure, g=(0,0,−g) is the gravitational acceleration, t is the time, and $S_{T}$ is the constitutive stress tensor. Two constitutive models were employed in this study to formulate a pseudoplastic and a viscoelastic model. These models were approximated by the built‐in Generalized Newtonian Fluid‐ (GNF) and Viscoelastic fluid frameworks, respectively. The GNF framework employed the Power‐law model, where the apparent viscosity of PDMS was implemented as described in the *Rheology* section. The GNF model neglected the memory effects of the material, by which the stress tensor was determined solely by the instantaneous deformation rate:

(5) $$S_{T}=2\mu_{\text{app}}\left(\dot{\gamma }\right)D_{T}$$

where $\dot{\gamma }=\sqrt{2\;\mathrm{tr}(D_{T}^{2})}$ is the strain‐rate magnitude, and the deformation rate tensor $D_{T}$ is defined as:

(6) $$\begin{array}{cc} D_{T} & =\frac{1}{2}\left(\nabla u+{\left(\nabla u\right)}^{T}\right) \end{array}$$

The viscoelastic material model employed the Giesekus constitutive equations to resolve the viscous and elastic properties of the material through the stress tensor formulation. The polymeric stress tensor $\tau_{p}$ evolves as:

(7) $$\lambda {\overset{\nabla }{\tau }}_{p}+\tau_{p}+\alpha \frac{\lambda }{\eta_{p}}\tau_{p}\cdot \tau_{p}=2\eta_{p}D_{T},$$

where, ${\overset{\nabla }{\tau }}_{p}$ is the upper‐convected derivative of $\tau_{p}$, λ is the relaxation time, α is the mobility factor (set to 0.5 to maintain mobility neutrality [19]), and $\eta_{p}$ is the polymer viscosity. The only distinction between the GNF and viscoelastic frameworks was the inclusion of the Giesekus model to account for the memory effects of the viscoelastic material, which enabled direct comparison between the pseudoplastic and viscoelastic fluid models under identical shear conditions. The relaxation time λ was numerically estimated from the oscillatory sweep data reported in the Larson study [16]. This limitation introduces uncertainty in the precise viscoelastic response but still allows for qualitative comparison of the different rheological regimes. The elastic stress was solved implicitly. An implicit successive under‐relaxation technique was used to compute the viscous stress in the momentum equation. Momentum advection was evaluated explicitly using a monotonically preserving upwind‐difference scheme, which ensured second‐order spatial accuracy and first‐order temporal accuracy. Pressure and velocity components were solved implicitly in time. Predictions of pressure and wall forces were facilitated by the immersed boundary method, which improved the estimation of extrusion pressure [20]. The time‐step size was initially set to $1\times 10^{-6}$, but it was subsequently adjusted dynamically by the solver based on stability criteria to avoid numerical instabilities [18]. The software employed the FAVOR (Fractional Area/Volume Obstacle Representation)[21] technique to represent solid geometries within the computational domain, enabling simulation of nozzle motion. Free‐surface tracking was performed using the Volume‐of‐Fluid (VOF) method; see Refs. [22, 23] for further details.

### 6.6 Validation of the Model: Quantitative Measurements

To validate the numerical model, sections of both experimental [16] and simulated strands were binarized, and their areas were compared using two similarity metrics, Dice and IoU (Figure 9). The analysis was performed for three different printing modes, all of which showed good agreement between experiments and simulations.

### 6.7 Effect of Relaxation Time on Patterning

To isolate the influence of viscoelasticity observed in the manually repeated simulations (Sec. Physics framework ‐ rheological considerations), a parametric study was performed in which the relaxation time λ was systematically reduced while keeping the input signal identical. As seen in Figure 10a, the deviations decrease with decreasing λ. For sufficiently small relaxation times, the creeping‐flow and history‐dependent effects are nearly eliminated, and the resulting microstructures closely resemble those obtained with the pseudoplastic model. This behavior is consistent with the logarithmic nature of viscoelastic relaxation (Figure 10b), wherein an incremental change in relaxation time Δλ must exceed a threshold before producing a measurable change in strain Δε. Given the more predictable flow response and the widespread use of shear‐thinning materials in extrusion‐based 3D printing, we therefore focused the subsequent development of our methodology on pseudoplastic materials, as complex microstructures in viscoelastic materials, especially at longer relaxation times, are difficult to program.

**FIGURE 10:**
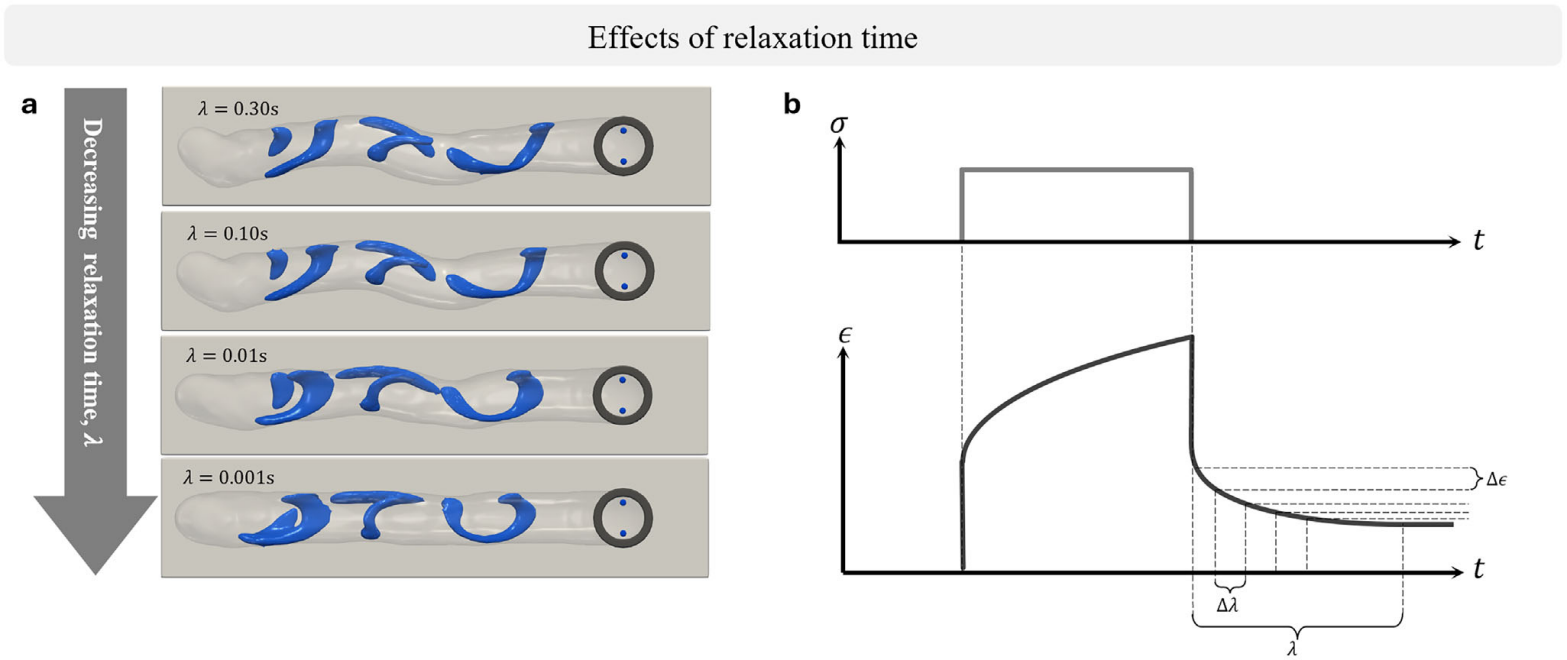
Effects of decreasing relaxation time on patterning.

### 6.8 µSlicer Optimization Loop

The optimization loop developed in this study, is inspired by the CHOMP [24] algorithm (Covariant Hamiltonian Optimization for Motion Planning) used in robotics to compute optimal robot trajectories through spatial domains with obstacle constraints. In contrast to its conventional use, where collisions are minimized, the present approach treats each particle as an obstacle to be intersected, reformulating the objective to maximize collisions instead. The optimizer works by iteratively calculating, for each timestep, i, the most optimal nozzle rotation angle $\theta_{i}\in \left[-\pi ,\pi \right]$, and the most optimal extrusion state of the outlets, $e_{i}=\left[e_{1,i},e_{2,i}\right]\in \left\{\left[0\;1\right],\left[1\;0\right],\left[1\;1\right]or\left[0\;0\right]\right\}$. Here, the extrusion state is defined as a binary variable, with a value of 1 denoting active material extrusion and a value of 0 denoting no extrusion, while the index indicating the corresponding outlet. As previously mentioned, it is the objective of the optimizer to maximize the number of target point hits. This objective can in extreme cases increase the chances of non‐physical rotations as well as jittery extrusion states. To minimize these effects, the algorithm applies penalties and rewards to enforce smooth rotational behavior and stable extrusion, penalizing large angular changes while rewarding target selections that preserve identical extrusion states. The first step of the developed optimization algorithm draws inspiration from the cost‐field formulation used in CHOMP, in which candidate paths are evaluated and the path with the minimum associated cost is selected. Rather than selecting between various paths, this algorithm chooses points with lowest cost. This is performed by constructing a field, $f_{j}$, covering the area of the nozzle, in which each target point, $\left(x_{j},y_{j}\right)$, is treated as a Gaussian kernel yielding the formulation (8) for the field.

(8) $$f_{j}\left(x,y\right)=\exp\left(-\frac{{\left(x-x_{j}\right)}^{2}+{\left(y-y_{j}\right)}^{2}}{2\sigma^{2}}\right)$$

However, as the current field formulation yields in high values close to the target points, the field is converted to a cost field, $C_{j}$, with the following formulation.

(9) $$C_{j}\left(x,y\right)=1-f_{j}\left(x,y\right)$$

This cost field formulation yields low cost near target points and higher cost as the distance from the target points increases. In addition, the cost field is bounded to values $C_{j}\left(x,y\right)\in \left[0,1\right]$. The next step is to compute a sequence of potential candidates hitting low cost areas of the cost field, while still being within the printers two outlets' reach. These are calculated depending on the nozzle position $\left(x_{n},y_{n}\right)$, its current nozzle attitude $\theta_{i}$, sampling resolution, N, and half angle, δ. First, the current angle for each orbiting outlet is calculated.

(10) $$\begin{array}{cc} \theta_{1,i} & =\theta_{i} \end{array}$$

(11) $$\begin{array}{cc} \theta_{2,i} & =\theta_{i}+\pi \end{array}$$

An array of potential angles, $\theta_{k}^{N}$, is constructed so that $\theta_{k}^{N}\in \left[\theta_{k}-\delta ,\theta_{k}+\delta \right]$. Note that the superscript, N, denotes the specific angular sampling, while the index k is either 1 or 2 depending on the specific outlet. A target point, $\left(x_{j},y_{j}\right)$ is considered hit by outlet k, if its angular coordinate, $\phi_{j}$, lies within the arc of potential angles in Equation (12) and its distance from the nozzle center, $d_{j}$, is within the outlet orbit path, r, with a given tolerance $\varepsilon \in \left[0,1\right]$ in Equation (13). For this study, a tolerance of ε=0.2 was chosen.

(12) $$|\phi_{j}-\theta_{k}^{N}|\leq \delta$$

(13) $$|d_{j}-r|\leq r\cdot \varepsilon$$

If both conditions are satisfied for the current potential angle, the hit evaluator parameter, $H_{k}$, is assigned a value of 1; otherwise, it remains 0, indicating no hit. The array of potential angles is then used to compute the candidate points, $\left(x_{k}^{N},y_{k}^{N}\right)$ given in Equation (14), that are then used to evaluate their associated costs using bilinear interpolation from the precomputed cost field e. g., $C_{k}\left(x_{k}^{N},y_{k}^{N}\right)$.

(14) $$\left(x_{k}^{N},y_{k}^{N}\right)=\left(x_{n}+r\cdot \cos\theta_{k}^{N},y_{n}+r\cdot \sin\theta_{k}^{N}\right)$$

The cost evaluation and hit detection is then used to evaluate the score, $S_{i,j}$, of each extrusion state scenario for each target point based on a the following approach given in Equation (15).

(15) $$S_{i,j}=\left\{\begin{array}{cc} \alpha H_{1}-\beta C_{1} & \text{For}\;e_{i,j}=\left[1\;0\right]\\ \alpha H_{2}-\beta C_{2} & \text{For}\;e_{i,j}=\left[0\;1\right]\\ \alpha \left(H_{1}+H_{2}\right)-\beta \left(C_{1}+C_{2}\right) & \text{For}\;e_{i,j}=\left[1\;1\right] \end{array}\right.$$

Here, α and β are weighting factors that regulate the trade‐off within the optimization process. Specifically, α governs how strongly the optimizer is driven to hit target points, effectively promoting solutions that maximize coverage or accuracy with respect to the desired locations. In contrast, β penalizes regions associated with higher cost, encouraging the optimizer to favor safer or more smoother paths. The relative magnitude of these weighting factors determines the overall behavior of the system. For instance, a high α combined with a low β may lead to aggressive strategies that prioritize hitting targets, even if this results in inefficient or risky paths. Conversely, a higher β shifts the balance toward conservative behavior, where the optimizer avoids costly regions, potentially at the expense of missing some target points. As such, careful tuning of α and β is essential to achieve a desirable balance between accuracy and robustness, depending on the specific objectives and constraints of the application. As previously mentioned, relying solely on a maximum score can result in extreme non‐physical rotations or jittery extrusion states. To address this, penalty terms are introduced for both rotations and extrusion states and subtracted from the score, $S_{i,j}$. The parameters $\lambda_{s}$ and $\lambda_{e}$ are used to control the penalties associated with rotational smoothness in Equation (16) and extrusion hysteresis (17), respectively.

(16) $$-\lambda_{s}\cdot {\left(\Delta \theta_{i}\right)}^{2}$$

(17) $$-\lambda_{e}\cdot ∥e_{i}-e_{i-1}∥$$

After applying the penalties, the extrusion state with the maximum score can be chosen for the current time step, by which its corresponding angle $\theta_{i}$ can be extracted and the optimized path signal can be constructed. Because the optimizer operates in a discrete manner, a Savitzky–Golay [25] filter is applied as a final post‐processing step to smooth the signal, ensuring compatibility with 3D printing requirements.

### 6.9 Selection of the Initial Target Point

Since each material outlet can target only one point at a given printing time, the algorithm must select which target point should be reached at each step. In the current implementation, this selection is sequential: when the outlet position is chosen to reach a target point at step n, the algorithm does not account for which target point should be reached at step n+1. However, the selection at step n+1 is constrained by the outlet position already chosen at step n. As a result, early decisions can strongly influence the subsequent trajectory, even though they are made without knowledge of the later points that must be reached. This makes the initial target point important, and in the current demonstrations this starting point was guided to avoid convergence toward unsuitable signal trajectories. A more globally optimized approach, in which the selection of each point considers both previous and future target points, could reduce this dependence on initialization, but would also increase computational cost.

### 6.10 Raytracing of Microstructure: What You See is Not What You Get

Visual inspection is widely used in multimaterial 3D printing to assess the internal microstructure of printed strands [16, 27, 28, 29]. This approach works well when the deposited materials are opaque, since their positions are directly visible on the strand surface. However, when the microstructure is embedded within a transparent matrix, the apparent morphology can be strongly distorted by optical refraction. This becomes particularly critical for subvoxel programmability, where the precise placement of microscale features is essential. If refraction shifts the perceived position of these features, visual inspection alone cannot reliably inform printing strategy or validate the underlying material arrangement. To address these optical distortions, the CFD‐predicted microstructures, as generated within the µSlicer framework, were post‐processed using progressive ray tracing together with a calibrated PDMS optical material profile used in experiments [16], enabling physically accurate rendering of refracted light paths.

Intel's OSPRay, integrated in ParaView, is a CPU‐based physically based ray tracing framework for scientific visualization. In contrast to conventional rasterization, OSPRay computes image formation by tracing rays from the camera through the computational domain, evaluating their interactions with surfaces and volumetric data. For volume rendering, a ray‐marching approach is employed, where scalar field values are sampled along each ray and mapped to color and opacity via transfer functions, with contributions accumulated to form the final pixel intensity. This approach enables realistic depiction of light transport effects, including shadows, transparency, and scattering, making it well suited for complex volumetric datasets such as the simulations of transparent PDMS material. The custom PDMS material profile was implemented with a refractive index of n=1.4 to reproduce the optical characteristics of the PDMS Sylgard 186 base used in the Larson et al. [16] experiments.

A systematic study was conducted in which the same CFD results were rendered with different refractive indices to quantify how refraction affects the perceived microstructure, as can be seen in Figure 11. For context, refractive indices of n≈1.0 correspond to air‐filled pores or aerogels [30]; n≈1.33 is representative of common highly hydrated hydrogels and soft bioinks such as PEG‐based or alginate systems (similar to water) [31]; n≈1.5 corresponds to typical printable polymers and resins, including PDMS, SLA/DLP photopolymers, PMMA, and polyurethane acrylates [32]; and refractive indices approaching n≈2.0 can occur in printable high‐index composites, such as resins or silicone loaded with titania (TiO_2_) or other high‐index ceramic nanoparticles [26].

**FIGURE 11:**
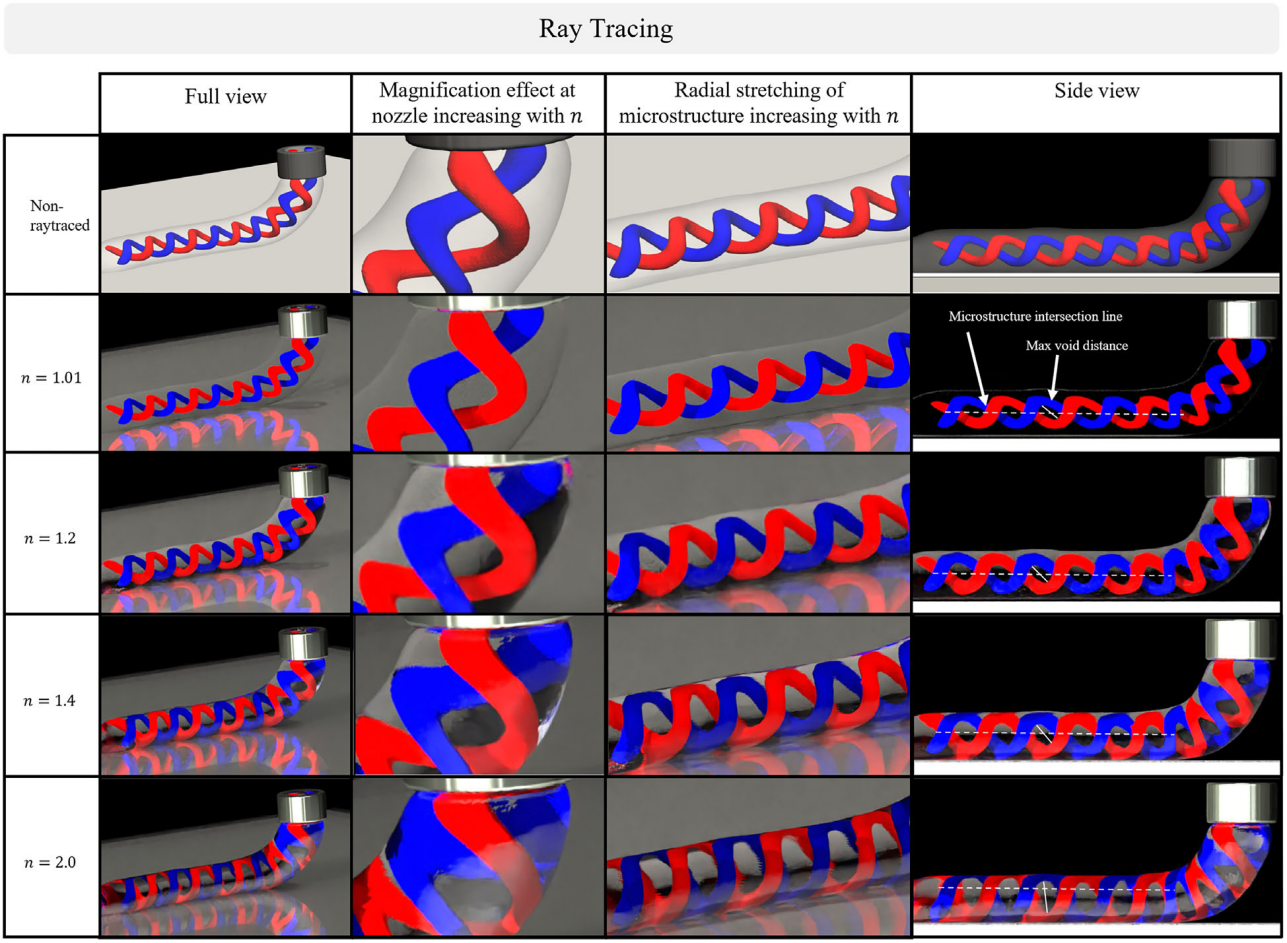
Appearance of the strand obtained by ray tracing into numerical results for different bulk material with distinct refractive indices. The refractive index was varied from n = 1 (air) to n = 2 (resins or silicone loaded with high‐index ceramic nanoparticles [26]).

Comparisons between the ray‐traced and non‐ray‐traced results, which represent the true material locations, revealed systematic shifts in the apparent microstructure with increasing refractive index. The effect was most pronounced directly beneath the nozzle outlet, where the blue‐dye region appeared increasingly magnified as n increased. In the deposited strand, this manifested as a radial stretching of the microstructure (Figure 11): the transparent regions between the red and blue dyes widened with increasing refraction, and for n≥1.4, the red and blue arches of the helical microstructure appeared to touch the strand sidewalls despite being physically separated in the CFD geometry.

These results highlight that optical refraction can significantly distort the visual appearance of embedded microstructures, an important consideration because many RM‐3DP experiments, including those of Larson et al., rely on imaging through transparent materials. Consequently, accurate comparison between experimental observations and numerical predictions requires accounting for refraction; otherwise, the apparent microstructure may differ substantially from its true physical configuration. Beyond this, the analysis underscores that refractive effects can themselves be harnessed: by precisely controlling subvoxel material placement, virtual RM‐3DP enables not only microstructural programmability for mechanical or thermal properties but also the intentional tuning of optical properties such as apparent feature position, contrast, and magnification within transparent matrices.

## Author Contributions

D.H.M, B.Š. and J.S. designed the research. D.H.M, B.Š. and J.S. performed the research. D.H.M, B.Š., T.M., Y.J., R.W. and J.S. analyzed the data. D.H.M, B.Š., T.M., Y.J., R.W. and J.S. prepared the manuscript.

## Conflicts of Interest

The authors declare no conflicts of interest.

## Data Availability

Data supporting the findings of this study can be obtained from the corresponding author upon reasonable request.
